# Obstetrics

**Published:** 1892-06

**Authors:** L. M. Griffiths


### OBSTETRICS.

German obstetricians, whose contributions to the science and art of midwifery are so many and so noteworthy, insist with much force upon the danger to the mother when the attendant makes frequent vaginal examinations. Dr. Julius Rosenberg [1] summarises these opinions, and endorses them with great emphasis. He pleads for the management of labour without internal examinations, which should be resorted to only in doubtful cases. He maintains that by abdominal palpation and auscultation we can say if labour has commenced, and can discover the position of the child and the presenting part, and the other conditions which denote the stage of the labour. Dr. Rosenberg says that if everything is found to be normal, it is only frivolous curiosity which can subject the woman to the danger of a vaginal examination. Many will, however, think that this is carrying an attempted prophylaxis needlessly far, and will doubt whether in those cases in private practice in which a

[1] *Med. Rec.*, Jan. 23, 1892.

condition of anæsthesia is not maintained, the maternal appeals for help could be usually satisfied by the negations of this method. Anyway, Dr. Rosenberg is right in demanding that the student should be more thoroughly taught the method of abdominal palpation than is commonly the case.

Dr. Rosenberg protests against the routine practice of vaginal douches, and from a large number of cases quotes figures to show that the mortality is increased where it is adopted.

Another shock to the security felt by the use of the perchloride of mercury as an antiseptic is conveyed to us by a communication from Dr. Howard A. Kelly,[1] of the Johns Hopkins University. Dr. Kelly, whilst acknowledging that instruments and dressings can easily be made thoroughly aseptic, considers that sufficient attention is not paid to the surgeon's hands. He made a series of sixty-five experiments, consisting of cultures taken from the epidermis of the hands both before and after using certain disinfecting agents. Germs developed freely after the application of a solution of the perchloride as strong as 1 in 500, but there was little or no development after the employment of permanganate of potash, which, if used in the way he recommends, Dr. Kelly considers to be the most efficient germicide in our possession. But the weak solutions upon which reliance is so commonly placed are powerless, and the salt must be used according to the following method:

(1) The hands must be scrubbed for ten minutes in water, frequently changed, at about 104° F. Special attention must be paid to the nails.

(2) The hands must be immersed in a saturated solution of the permanganate until every part of the hands and lower forearms is stained a deep mahogany or almost black colour. They are then transferred at once to a saturated solution of oxalic acid until completely decolourised, and of a healthy pink colour.

(3) The oxalic acid is to be washed off in warm sterilised water.

Dr. Kelly says that by this simple process the hands are rendered more nearly absolutely aseptic than by any other known means.

For gynæcological or other operations this process may

[1] *Amer. Journ. Obst.*, Dec., 1891, p. 1414.

perhaps be called "simple," but it is doubtful if it is practicable in ordinary midwifery work. Happily Dr. Kelly speaks well of the disinfecting powers of soap and water freely used, and allows that the perchloride inhibits further growth of the coccus until the salt is precipitated or removed, and for this accoucheurs must be grateful.

An authoritative code of "Rules for the Use of Antiseptics in the Practice of Out-door Maternities"[1] has been issued by the committee of the General Lying-in Hospital, York Road, Lambeth, and might with advantage be studied and remembered by all engaged in the practice of midwifery.

Most practitioners are alarmed when they find a considerable rise in the temperature of a woman recently confined; and they begin to fear that it is the beginning of a septic trouble which may not improbably end in death, or, at best, in a tardy convalescence. But in many cases a condition of pyrexia is transient, and not the result of wound-infection or of dangerous complication. Dr. J. Chalmers Cameron[2] points out, with much clearness and care, that temporary elevation of temperature may be caused by emotional disturbances, or reflex irritation; and he gives many practical details, attention to which may save the attendant much needless anxiety.

On the subject of puerperal septicæmia, there were two interesting papers at the meeting of the British Gynæcological Society at Newcastle-on-Tyne last year. Dr. Oliver narrated his experience of twenty-five cases which he had seen in the preceding eighteen months; and Dr. Dolan gave the results of his practice, which consisted of 3,453 cases of confinements attended without antiseptic precautions, and in which there were only two cases of septicæmia. The papers in full, and the discussion which followed, are reported in the *British Gynæcological Journal* for November, 1891.

Dr. J. A. G. Hamilton,[3] reviewing 1,000 consecutive cases in his practice from 1883 to 1890, said that he had had no case of septicæmia, and that he had not adopted any elaborate antiseptic precautions beyond cleanliness and boiling of any instruments used. In all cases in which it had been necessary to introduce the hand into the uterus he had washed out the cavity with some antiseptic solution.

[1] *Brit. Med. Journ.*, Feb. 6, 1892, p. 313.

[2] *Internat. Clinics*, vol. i., 1891, p. 164.

[3] *Australas. Med. Gaz.*, April, 1892, p. 183.

Mr. Melville Jay[1] records notes of the last thousand cases he had attended. One death from septicæmia had occurred. The only antiseptics he used were hot water for hands and instruments and an antiseptic lubricant. When the labour is over he thoroughly cleanses the patient.

Dr. N. W. Rand[2] considers that the old plan of preserving the perineum by digital dilatation of the vaginal orifice has been too much neglected. He believes that, if given a fair trial by using two fingers and making steady traction back toward the coccyx, it will be found more efficient than any of the other methods that have been suggested, and infinitely less unpleasant to doctor and patient than the much-vaunted practice of directing the head forwards by means of two fingers in the rectum. But Dr. Rand's advice violates the principles of the antiseptic treatment, and cannot be recommended.

Dr. William Goodell,[3] discoursing on a case in the Clinic of the Hospital of the University of Pennsylvania, demonstrated the importance of thorough intra-uterine dealing with the placenta in cases of miscarriage. Six months before the patient came under treatment she had a miscarriage at three months, and from that time had a constant, but inodorous, bloody discharge, with several severe hemorrhages, producing marked constitutional effect. With strict antiseptic precautions, the interior of the uterus was explored, and it was then shown that the curette, useful as it is for scraping off fungous vegetations and other small growths, was an inefficient instrument for the separation even of the small attached placenta, which was then removed by a half-twist with a small fenestrated polypus-forceps. The bleeding was stopped by the injection of a syringeful of hot vinegar. Dr. Goodell lays stress on the duty of removing the placenta at the time of the miscarriage, and thinks this is best done either by the forceps or the index-finger.

The profession is not, however, of one opinion as to the treatment of abortion. The subject was discussed in the New York Academy of Medicine[4] on November 18th, 1891. Dr. H. G. Locke described the practice at Roosevelt Hospital, in which nothing is left to Nature, but an operation for

[1] *Australas. Med. Gaz.*, April, 1892, p. 188.
[2] *Med. Rec.*, Dec. 5, 1891, p. 677. [3] *Internat. Clinics*, vol. i., 1891, p. 190.
[4] *Med. Rec.*, Jan 16, 1892, p. 78.

emptying the uterus is carried out with all the precautions of a major abdominal operation. Dr. R. A. Murray thought the treatment too complex for private practice. He considered that, short of hemorrhage or sepsis, there was no need for interfering with the uterus. He deprecated the hospital training of men in methods which they could not use in outside practice. Other speakers considered intra-uterine treatment uncalled for in most cases.

Not only is there usually grievous domestic disappointment about abortions, but the health of the mother is often seriously undermined. It behoves the practitioner to thoroughly investigate the causes of an abortion, in order, if possible, that another one may be prevented. Schuhl has given considerable attention to this question (*Annales de Gyn.*, May, June, and July, 1891).[1] Among the very numerous etiological factors of repeated abortion, he considers that uterine affections—especially retroflexions—and syphilis are of especial importance.

Following a paper by Dr. Gustav Zinke, before the Cincinnati Academy of Medicine, on "Two Cases of *Post-partum* Hemorrhage," there was an interesting discussion,[2] in which the balance of opinion was against tamponading the uterus with iodoform gauze, although the practice has received commendation by high authority. Some speakers bore strong testimony to the value of vinegar in restraining hemorrhage that occurs after removal of the placenta, and recommended that a tumbler of vinegar should be always within reach, and that a handkerchief or some strips of muslin should be dipped in it, and, if need be, passed into the uterus.

The work of inducing respiration in cases of pronounced asphyxia in the new-born infant is often tedious and disappointing. Dr. W. E. Forest[3] of New York says that of the various methods recommended only three can be shown, by careful trial and experiment, to be of any practical service in introducing an appreciable amount of air into the collapsed lungs. These are: (1) Sylvester's well-known method; (2) Schultze's swinging method, to which reference was made in the *Journal* for June, 1891; and (3) the introduction into the trachea of a catheter, by means of which air is blown in

[1] A very full abstract of his communication is given in the *Amer. Journ. Obst.*, Feb., 1892, to which reference should be made for details.
[2] *Cincin. Lanc.-Clin.*, Feb. 13, 1892, p. 212. [3] *Med. Rec.*, April 9, 1892.

directly from the mouth or the bellows. Dr. Forest reviews in detail the advantages and disadvantages of these methods, and concludes that in bad cases neither of them can be trusted. He considers that the plan which he has practised in some cases that he records is not only efficient, but is free from the dangers incidental to the other methods. His directions are as follows:

1. Lay the child on its face for an instant with the head and thorax lower than the pelvis, and make quick, but not violent, pressure on the child's back. This is done to expel any fluids that may have been drawn into the child's mouth while passing through the pelvic canal.
2. Place the child in the sitting posture in a pail or tub containing six to eight inches of water, as hot as can be borne comfortably by the operator's hand. The child is supported in this position by one of the operator's hands across the child's back, the child's head bent back, and resting in the crotch between the thumb and forefinger of this hand. The child's hands, with the palms to the front, are held in the other hand of the operator.
3. The child's hands are carried upward until the child is suspended by the arms, the buttocks just raised from the bottom of the pail. The child's head now falls back, and the operator leans forward, and, mouth to mouth, blows into the child's lungs.
4. The child's arms are then lowered until the hand of the operator holding them rests across the front of the child's thorax. Then the body of the child is doubled forward, and, at the same time, its thorax is compressed between the operator's hands—one in front, the other behind. This expels the air from the lungs, and completes the movements.

The profession in the Leeward Islands has for some years been striving to get an improved class of midwives, and has, from time to time, appealed to the authorities to secure this, but hitherto without success. Last year Mr. A. P. Boon[1] brought the question before the recently-formed Leeward Islands Branch of the British Medical Association, which he easily persuaded to pass the following resolution: "That this branch of the British Medical Association is of opinion that provision should be made for the proper educa-

[1] *Leeward Islands Med. Journ.*, 1891, p. 121.

tion, licensing, and registration of the midwives in this colony, whereby the mortality among lying-in women and their infants would probably be much reduced."

Last year also the North Queensland Medical Society, after hearing a paper by Dr. W. B. Nisbet on "The Education of Midwives,"[1] unanimously resolved: "That the time has arrived when the indiscriminate practice of midwifery by untrained nurses should be discouraged in the towns in the colony, and provision should be made for educating suitable women to become certificated midwives." From India comes the same note. Mr. P. S. Mootooswamy, of Tanjore, recording some "Contributions to the Practice of Midwifery,"[2] takes occasion to put in a plea for educated and trained midwives to supplant ignorant and untaught women in the attendance on women belonging to the poor and labouring classes in that country.

The mother country must not be behind its colonies in safeguarding the life and health of the class of women who will, so far as can be seen at present, have to be always attended by midwives, and Parliament could scarcely do anything more practically useful than pass a well-considered Act which would prevent women taking up the practice of midwifery till they had shown to some competent authority that they possessed the knowledge necessary for the work.

It is satisfactory to note the appointment of a Select Committee on the Midwives' Registration Bill now before the House of Commons. Although this Committee began taking evidence[3] on May 13th, the state of public business does not promise much real progress with the question. Less important matters must first be settled.

L. M. Griffiths.

### LARYNGOLOGY AND RHINOLOGY.

[1] *Austral. Med. Gaz.*, June, 1891. [2] *Ind. Med. Gaz.*, August, 1891.

[3] *Brit. Med. Journ.*, May 21, 1892, p. 1092.

